# Strategies to enhance remote monitoring adherence among patients with cardiovascular implantable electronic devices

**DOI:** 10.1016/j.hroo.2023.11.002

**Published:** 2023-11-08

**Authors:** Thomas L. Rotering, Sylvia J. Hysong, Katherine E. Williams, Merritt H. Raitt, Mary A. Whooley, Sanket S. Dhruva

**Affiliations:** ∗ San Francisco Veterans Affairs Health Care System, San Francisco, California; † Section of Cardiology, Department of Medicine, University of California, San Francisco School of Medicine, San Francisco, California; ‡ Philip R. Lee Institute for Health Policy Studies, University of California, San Francisco, California; § Center for Innovations in Quality, Effectiveness, and Safety, Michael E. DeBakey VA Medical Center, Houston, Texas; ‖ Section of Health Services Research, Department of Medicine, Baylor College of Medicine, Houston, Texas; ¶ Department of Medicine, University of California, San Francisco School of Medicine, San Francisco, California; ∗∗ Portland Veterans Affairs Health Care System, Portland, Oregon; †† Knight Cardiovascular Institute, Oregon Health and Sciences University, Portland, Oregon; ‡‡ Division of General Internal Medicine, Department of Medicine, University of California, San Francisco School of Medicine, San Francisco, California

**Keywords:** Cardiovascular implantable electronic device, Pacemaker, Implantable cardioverter-defibrillator, Remote monitoring, Patient adherence

## Abstract

**Background:**

Remote monitoring (RM) of patients with cardiovascular implantable electronic devices (CIEDs) (pacemakers and implantable cardioverter-defibrillators) has a Class 1, Level of Evidence A Heart Rhythm Society recommendation. Yet RM adherence varies widely across settings, and factors associated with variation are not understood.

**Objective:**

The purpose of this study was to identify strategies for supporting RM across Veterans Health Administration (VHA) facilities.

**Methods:**

In a national evaluation, we surveyed and interviewed 27 nurses, medical instrument technicians, and advanced practice providers across 26 VHA facilities (following approximately 15,000 CIED patients). Participants were selected based on overall patient adherence by facility, which ranged from 46%–96%. Questions covered RM adherence strategies, manufacturer resources, organizational characteristics, and workflows for optimizing adherence.

**Results:**

All clinicians reported that RM adherence was extremely important (53.8%), very important (34.6%), or important (11.5%) for improving patient outcomes. High performing facilities prioritized consistent patient education about RM and evaluated nonadherence using dashboards and manufacturer web sites. High performing facilities instituted clear standard operating procedures that defined staff responsibilities and facilitated efficient contact with nonadherent patients and then family members by phone and then mail. Clinicians based at high performing facilities spent twice as many hours per week (9.1) on average managing RM adherence compared to other facilities (4.5). Effective communication (internally and with non-VHA care partners) and use of CIED manufacturer resources were essential. Facilities that were not high performing rarely used these strategies.

**Conclusion:**

Clinicians can support high RM adherence by emphasizing patient education, regularly assessing and addressing nonadherence using staff protocols, and engaging CIED manufacturers.

## Graphical abstract

**Figure:**
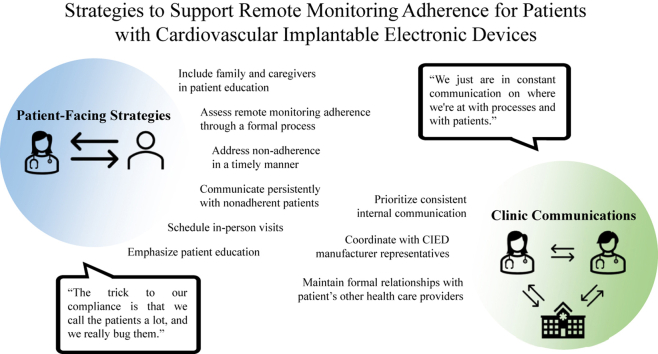

**Key Findings**

▪ Adherence to remote monitoring is essential for patients with cardiovascular implantable electronic devices (CIEDs) to receive the evidence-based benefits of this Class 1, Level of Evidence A Heart Rhythm Society recommendation.
▪ Facilities that had the highest proportion of patient adherence to remote monitoring prioritize consistent patient and caregiver education and use dashboards to evaluate nonadherence.
▪ High performing facilities also use standard operating procedures to enhance staff effectiveness to improve adherence.
▪ High performing facilities have a higher number of full-time staff equivalents to care for patients with CIEDs.

## Introduction

Remote monitoring (RM) enables patients with cardiovascular implantable electronic devices (CIEDs) (pacemakers and implantable cardioverter-defibrillators [ICDs]) to transmit data about arrhythmias and device parameters from their residence through a home monitor. These data often inform clinical decision-making, and RM has been demonstrated to reduce mortality,^1^, ^2^, ^3^, ^4^ inappropriate shocks among patients with ICDs,^5^^,^^6^ emergency department visits,^7^ hospitalizations,^4^^,^^8^^,^^9^ and health care costs.^7^^,^^10^ RM also reduces the number of outpatient follow-up visits^8^^,^^11^^,^^12^ and produces high levels of patient satisfaction and acceptance.^13^^,^^14^ Accordingly, RM has a Class 1, Level of Evidence A (strongest) professional society recommendation.^15^^,^^16^

Despite the evidence-based benefits of RM to patients and clinicians, RM adherence is suboptimal.^3^^,^^17^ Adherence has been found to be lower among patients with more advanced age, Black or African American patients, Hispanic or Latino patients, those with lower income, and in different geographic areas.^17^ Because patients receive the clinical benefits of RM only if they send regular transmissions and remain connected, there is a need to improve RM adherence. However, device clinicians who can support patient adherence face multiple challenges and competing priorities, including high alert volume, difficulty in demonstrating productivity, and lack of staffing.^18^

**Figure 1:**
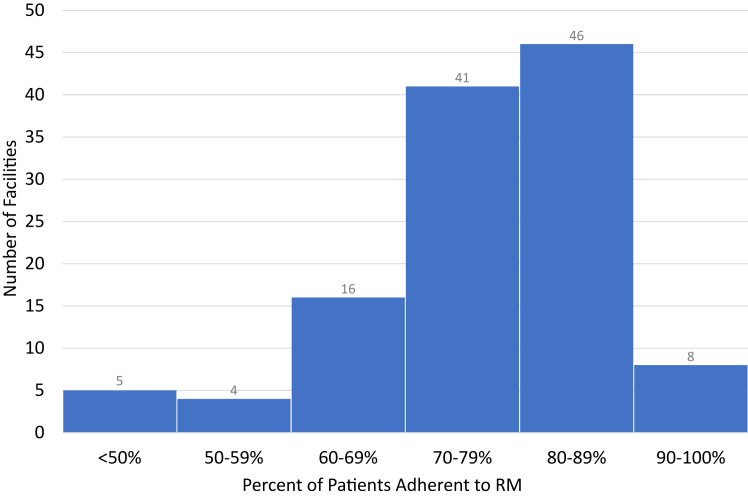
Number of Veterans Affairs facilities that provide care for patients with cardiovascular implantable electronic devices by percentage of patients adherent to remote monitoring (RM).

Research focused on patients with CIEDs has found that some patients may have limited understanding of RM, which is associated with lower adherence.^19^ Focus group research among patients with ICDs who were both adherent and nonadherent identified that an early and thorough explanation of RM purpose and function was important for adherence.^20^ However, there has not been a systematic examination of clinician-level strategies to support RM adherence. The Veterans Health Administration (VHA) provides an ideal opportunity to study barriers and facilitators to supporting adherence. Within the VHA, 122 unique facilities serve more than 60,000 veterans with CIEDs who participate in RM. There is substantial variation in patient adherence across facilities (46%–96%) (Figure 1), but reasons for this variation—why some facilities do well in supporting RM adherence while others do not—is unclear. Therefore, to understand which RM clinical practices make facilities successful, we conducted a national evaluation of 26 VHA facilities that care for approximately 15,000 patients with CIEDs.

## Methods

Between March and September 2022, we surveyed and interviewed a purposive sample of 27 clinicians (registered nurses, medical instrument technicians, and advanced practice providers) who lead RM efforts for veteran patients with CIEDs at their facility. Our goal was to identify barriers and facilitators to supporting RM adherence and to synthesize successful strategies. To ensure methodological rigor in study execution and subsequent reporting, we followed the COREQ (COnsolidated criteria for REporting Qualitative research) reporting standards (see Supplemental Material for checklist). We also sent a follow-up e-mail inquiry to all 27 clinicians in August and September 2023 to clarify facility staffing and time spent on RM and other device-specific care at the time of their interview (78% response rate).

### Setting and context

This quality improvement project was based on a partnership between the VHA National Cardiac Device Surveillance Program (VANCDSP) and Measurement Science Quality Enhancement Research Initiative (QUERI). In accordance with the Department of Veterans Affairs Office of Research & Development Program Guide 1200.21 “VHA (Veterans Health Administration) Operations Activities That May Constitute Research,” data were collected as part of a quality improvement study to assess and improve the quality of RM care for veterans with CIEDs and did not require institutional review board approval.

All willing and eligible patients with CIEDs followed by VHA facilities must be offered RM via the VANCDSP. The VANCDSP provides RM for patients with all varieties of CIED models and devices. These CIEDs include both those that have transmitters that require patient interaction and those that automatically send transmissions (approximately 85% of the VANCDSP patient population), which are associated with higher adherence.^17^ All remote transmissions are reviewed centrally by trained readers, who alert local facilities of findings that could be clinically relevant. Some VHA facilities do not have capability to implant CIEDs; these facilities still follow patients implanted at other facilities. Each remote transmission is available to local clinicians through VHA web-based platforms and CIED manufacturer web sites.

### Conceptual framework

Successful RM requires interaction and coordination among multiple parties, including the clinical team, the health care facility, the patient, and the CIED manufacturer, each of which can be the source of multiple factors driving RM adherence success. Consequently, we used the 2022 updated Consolidated Framework for Implementation Research (CFIR) framework to guide our work.^21^^,^^22^ CFIR proposes 5 general domains (and corresponding subdomains) of factors driving implementation success: the innovation itself (in this case CIED RM); the inner setting (the setting in which the innovation is implemented); the outer setting (the larger setting in which the inner setting exists); individuals (their roles and characteristics); and the implementation process. For this study, we focused on the inner setting and used CFIR inner setting subdomains to construct our survey and interview guide. Our analysis qualitatively characterizes consistent themes in clinical strategies.

### Participants

Clinicians were selected based on RM adherence rates at their facility and their leadership role in their facility’s RM operations. At each facility, nursing staff were primarily responsible for RM adherence. Interviews were conducted to include facilities with high performance on RM adherence (≥90% of patients with a CIED followed by that facility had sent a remote transmission in the past 100 days), normal/target range adherence (70%–89.9%), and suboptimal adherence (<70%) (Supplemental Table 1) (see Supplemental Table 2 for 100-day and 200-day adherence rates). Veterans served at each facility are scheduled to send transmissions at least every 90 days, with a 10-day buffer before being considered nonadherent. Clinicians in facilities serving a smaller number of patients (<250 patients) were also purposively sampled given that they may have different resources than larger facilities.

### Procedure

Clinicians were e-mailed (from authors TLR and SSD) at least 3 requests to participate in a voluntary survey and interview over multiple weeks. Recruitment was aided by the relationship between the NCDSP and clinicians established before study commencement, and clinicians were given background information about the objectives. Consent to interview was obtained via e-mail, and consent to audio recording was obtained verbally before the interview.

#### Web-based survey

Before the interviews, participants were e-mailed a 19-question, web-based survey covering participant professional duties (eg, professional role, time spent on RM, years in current role), perspectives on RM adherence, tools to support adherence, and demographics (see Supplemental Material). Guided by CFIR, these surveys allowed interviewers to target questions based on provider’s reported clinical practice, prompted interviewees to consider dynamics of RM before the interview, and contextualized interview responses through multiple-choice, Likert scale, and open-response questions.

#### Interviews

We used CFIR to develop a 10-question, semistructured interview guide. The guide contained questions about clinician perspectives, experiences, and practices regarding RM initiation; assessment of patient adherence; and strategies for addressing nonadherence. Interviews were conducted by authors TLR and SSD, who also asked about internal and external organizational dynamics, including relative importance of RM compared to other tasks, leadership engagement in RM, communication with non-VHA partners and manufacturers, changes in facility workflow over time, strategic goals for RM adherence, beliefs and attitudes about RM, and desired support for RM adherence (see Supplemental Material).

We conducted a single 30- to 60-minute interview with each participant via a video conferencing platform, recording audio and transcript through built-in features. Other than the study team members and clinician interviewees, no other persons were present for interviews. Interviewer notes and recordings were synthesized into a detailed account of each interview. All records were kept on secure VHA servers, and transcripts were anonymized.

### Data analysis

The 5 domains of CFIR were used as the basic scaffolding for our content analysis, with a focus for successful strategies in the inner setting domain.^22^

Analysis was conducted by a multidisciplinary team working to improve NCDSP operations, including a cardiologist (SSD), PhD research health scientist (SJH), and public health research expert (TLR). TLR supplemented interview accounts with open-response information from surveys and calculated descriptive statistics to analyze survey responses. SJH and TLR analyzed interview accounts using ATLAS.ti 22 (ATLAS.ti Scientific Software Development GmbH, Berlin, Germany).^23^ CFIR constructs were used to construct an initial codebook, with additional codes added as more themes were identified. SH and TLR primary-coded 3 interview accounts and then secondary-coded each other’s accounts. Disagreements were resolved by consensus or, in rare cases, with input from SSD when related to clarification of CIED-specific care. Subsequent analysis was completed by TLR. Thematic saturation was tracked and calculated retrospectively using the saturation ratio of Guest et al,^24^ using a base size of 5 and a run length of 3. After all interviews were completed, we observed a saturation ratio of 7%, just slightly larger than Guest’s proposed new information threshold of 5%.

We also plotted the relationship between average full-time staff equivalents (FTE), average FTE per 1000 patients, and facility level of performance on adherence. Additionally, we ran a series of Spearman correlations to assess the relationship between facility-level patient adherence to RM and FTE per 1000 patients by task (total FTE, FTE spent on RM, and FTE spent on other device-specific tasks).

## Results

### Demographics

We requested interviews with 39 clinicians and interviewed 27 (response rate 69%), who represented 26 facilities, 8 of which were high performing. Of the 27 interviewees, 24 (89%) also responded to the survey (Supplemental Table 3). Survey respondents were registered nurses (12), advance practice providers (7), and medical instrument technicians (5). Four respondents had 10+ years of experience working with the VHA device clinic, and 7 had 6–10 years.

### Successful strategies

#### Patient education

Interviewees explained that initiation of RM requires consistent education and engagement of patients before CIED implant, before discharge, at wound check, and at subsequent visits (Table 1). Essential elements of this conversation, often including written take-home materials for patients, included the importance of maintaining RM adherence, CIED function and procedure for manual transmission, and the role of the device clinic. CIED manufacturer representatives often assisted with patient education. For some high performing facilities, education sometimes involved setting up the home monitor at discharge or at the wound check visit and sending a “handshake” transmission. Although some patients had privacy concerns about RM, lacked stable housing and Internet service, and/or were not facile with RM technology, high performing facilities often overcame some of these barriers through dedicated education, assistance from patients’ social support systems (as able), and adapting support within the patient’s context and to the patient’s needs.

**Table 1:** Strategies to support remote monitoring adherence for patients with cardiovascular implantable electronic devices

Strategies	Description
Patient-facing strategies	
Emphasize patient education	Educate patients before implant Communicate to patients that the expectation is adherence to RM Test RM with “handshake” transmission Repeat education and check for RM adherence at wound check and subsequent in-person follow-up visits
Include family and caregivers in patient education	Educate caregivers and/or family members at time of implant and subsequent visits Explain RM technological requirements to caregivers and/or family members
Assess RM adherence through a formal process	Set aside time outside of routine patient visits to identify patients who are nonadherent to RM Use digital tools, including dashboards and manufacturer web sites, to assess RM connectivity and adherence
Address nonadherence in a timely manner	Address nonadherence within, at most, 1 month of a missed transmission
Communicate persistently with nonadherent patients	Use phone, letter, and even certified letter if necessary Leverage communication with the patient’s support system if necessary (family members, friends, neighbors, home caregivers)
Substitute in-person visits	Increase in-person visits for those patients who are persistently nonadherent to RM
Clinic communications	
Prioritize consistent internal communication	Document standard operating procedures for assigning specific staff responsibilities and creating systems/workflows for review Document all patient communication about RM adherence in electronic health record and/or internal tracking documents to optimize efficiency
Coordinate with CIED manufacturer representatives	Coordinate with CIED manufacturer representatives to support RM education, initiation, and adherence Use, and encourage patients to use, CIED manufacturer troubleshooting services
Maintain formal relationships with non-VHA health care providers	Develop formal relationships with non-VHA care partners that implant CIEDs Communicate with non-VHA care partners to ensure continuous patient care and prevent loss to follow-up

CIED = cardiovascular implantable electronic device; RM = remote monitoring; VHA = Veterans Health Administration.

#### Family and caregiver support

In addition to the patient, clinicians based in high performing facilities described how family members and caregivers were often prioritized in CIED education to strengthen social support for RM. Family and caregiver engagement was highlighted as particularly important for patients who struggled to understand or use RM technology. This registered nurse described her high performing facility’s process:

“We have a conversation at their clinic appointment to discuss getting a device implanted. We start the conversation at that time regarding remote monitoring and then…we talk to them before the surgery. We talk to the family with the veteran that's going to be receiving the device…and again after surgery and again before they discharge the following morning. Then we go through and show them exactly how remote monitoring works. We even pair their devices with the remote monitor when we have those monitors in our clinic before they discharge.…The more that they're educated about it and understand the importance, I think that helps with the compliance.”

#### Scheduled review using digital tools

Thirteen clinicians (54%) (including all but 1 in the high performing category) reported dedicating regular time to assess patient population RM adherence using digital tools in their survey (Table 2). These tools most commonly included cloud-based VHA dashboards that tracked RM transmissions and CIED manufacturer web sites. Some clinicians also used Outlook (Microsoft, Redmond, WA) calendar reminders and Excel (Microsoft) spreadsheets with data from the dashboards, manufacturer web sites, and electronic health records. One registered nurse at a high performing facility reported that this was a weekly task:

“[One] person monitors every transmission that comes through, not just the alerts and then we can see who's been disconnected, who's missed.…I have an Excel file on the company, the patients, their last transmission, next transmission, next in clinic.…We go through at least once a week.…If they’ve missed a remote transmission, we call and let them know, especially if they’re disconnected on the wireless ones….”

**Table 2:** Survey results

Question	High performing facility (n = 9)	Normal-to-target performing facility (n = 12)	Suboptimal performing facility (n = 3)	Total (n = 24)
When do you address remote monitoring adherence (making sure patients transmit when they are supposed to and troubleshooting missed transmissions)?				
Never	0	0	0	0
Just when I see patients in clinic and check to see if they have transmitted	1	6	1	8
I set aside some time every day/week/month to look at people that have not transmitted and try to get them transmitting	8	3	1	12
Other	0	3	1	4
On average, approximately how many hours per week do you spend focused on remote monitoring adherence (making sure patients transmit when they are supposed to and troubleshooting missed transmissions)?∗	9.1 (5.7)	4.5 (5.4)	4.7 (4.6)	6.3 (5.7)
How important is remote monitoring adherence to improving outcomes for patients with CIEDs?				
Extremely important	8	5	1	14
Very important	1	5	1	7
Important	0	2	1	3
Slightly important	0	0	0	0
Not at all important	0	0	0	0
What tools do you use to support remote monitoring adherence among veterans in your clinic? (check all that apply)				
NCDSP dashboard	6	11	3	20
NCDSP patient registration application	6	9	2	17
Monitor company sites	9	10	2	21
Other	1	1	0	2
Which do you prefer to support your remote monitoring adherence efforts?				
NCDSP dashboard	5	3	2	10
NCDSP patient registration application	1	2	0	3
Both	2	7	1	10
Other	1	0	0	1
When do you generally contact patients about a missed transmission?				
<1 month after missed transmission	4	2	0	6
1– 3 months after missed transmission	4	4	1	9
4–6 months after missed transmission	1	2	1	4
7–12 months after missed transmission	0	0	0	0
I usually do not contact patients who have missed transmissions	0	4	1	5

CIED = cardiovascular implantable electronic device; NCDSP = National Cardiac Device Surveillance Program.

A full version of the survey can be found in the Supplemental Material.

∗ Values are given as mean (± SD).

Of the 15 clinicians based at normal/target or suboptimal performing facilities, 10 reported only checking adherence when preparing for scheduled in-person visits for CIED interrogation or receiving an alert transmission (Table 2).

#### Timely follow-up

**Figure 2:**
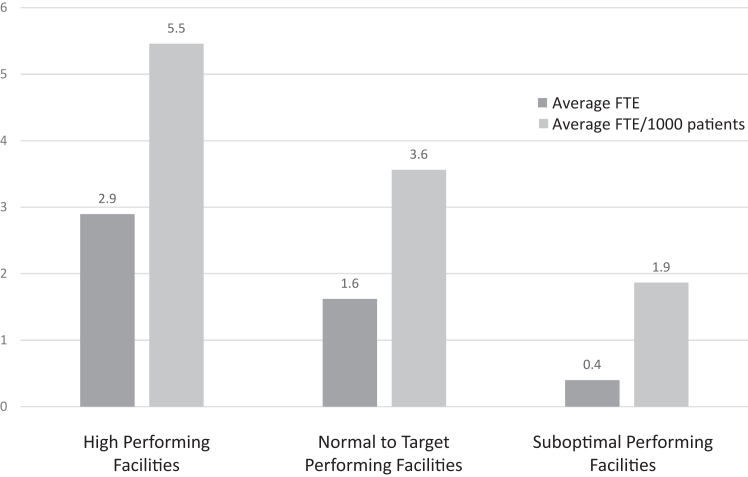
Average full-time staff equivalents (FTE) by facility level of performance.

Survey results indicated that clinicians in high performing facilities maintained close contact with all veteran patients with a CIED, particularly those who had become nonadherent to RM, contacting patients within <1 month (4/9) or 1–3 months (4/9) of a missed transmission (Table 2). In contrast, only 7 of 15 normal-to-target range and suboptimal performing facilities reported contacting patients within 3 months, with 5 of 15 not contacting patients at all. These efforts required considerable time. Respondents based at high performing facilities reported spending an average of 9.1 hours per week on RM adherence. In contrast, other facilities spent an average of 4.5 hours on RM adherence. Additionally, high performing facilities reported an average of 5.5 FTE per 1000 patients spent focused on care for veterans with CIEDs, nearly triple the FTE per 1000 patients at suboptimal performing facilities (1.9) (Figure 2 and Table 3). These tasks required a high degree of staff technical competence, supported by robust training and education, and included scheduling in-person follow-up appointments for patients who had fallen out of adherence. Spearman correlation showed a moderate positive correlation between total FTE and patient adherence to RM, which was statistically significant (r_s_ = .53; *P* = .019) (Supplemental Table 4). Additionally, there was a stronger positive correlation between FTE spent on RM and patient adherence to RM, which was statistically significant (r_s_ = .67; *P* = .002). However, there was a weakly positive correlation between FTE spent on other device-specific care of veterans with CIEDs and patient adherence to RM, which was not statistically significant (r_s_ = .25; *P* = .283).

**Table 3:** Average FTE per 1000 patients by facility performance level

	High performing facility	Normal-to-target performing facility	Suboptimal performing facility
	(n = 6)	(n = 12)	(n = 2)
Average patient count	629	583	211
Total FTE	5.5	3.6	1.9
FTE by title			
MD	0.8	0.2	0.0
APP, NP, or PA	1.6	1.1	0.2
RN or LPN	3.1	1.9	1.7
MIT or technician	0.0	0.3	0.0
FTE by task			
Remote monitoring∗	2.3	1.0	1.1
Other device-specific care of veterans with CIEDs†	3.1	2.5	0.7

APP = advance practice provider; CIED = cardiovascular implantable electronic device; FTE = full-time equivalent (40 hours per week); LPN = licensed practice nurse; MIT = medical instrument technician; MD = medical doctor; NP = nurse practitioner; PA = physician assistant; RN = registered nurse; other abbreviations as in Table 1.

∗ Remote monitoring = enrolling patients for RM, transferring patients to/from VA clinic, ordering patients monitors, following up on adherence/connectivity, addressing RM findings by phone, entering electronic health record notes on RM findings.

† Other device-specific care = in-clinic visits, preparation for in-clinic visits, follow-up visits in-person for RM findings, inpatient or emergency department consults, reprogramming devices for magnetic resonance imaging, patient education before or after CIED placement, excludes device implant.

#### Patient and caregiver communication

When a patient became nonadherent to RM, phone calls were attempted repeatedly as a first-line approach and often involved patient education or troubleshooting. These as-needed calls sometimes took substantial time, often lasting 30 minutes or longer. When clinicians could not contact patients by phone, some sent a letter and one even followed with a certified letter posted to the patient’s home address detailing the attempts to contact the patient, the need for RM, and the device clinic’s phone number.

Although contacting nonadherent patients and addressing their unique needs are essential components of CIED care, because it does not necessarily generate workload credit in the same way that an in-person visit or transmission review would, funding support for this clinician time was not guaranteed. Surveys indicated that most clinicians in every performance category (21/27) had difficulty contacting patients due to a lack of facility staffing. However, high performing facilities more often had adequate resources to hire additional clinical care team members with protected time to contact patients who had fallen out of RM adherence and assist in troubleshooting.

Describing efforts to address nonadherence, one registered nurse at a high performing facility stated:

“The trick to our compliance is that we call the patients a lot and we really bug them.…Compared to some clinics I've talked to; we have the staff and we've been given the FTE [full-time equivalent] to be able to have the time that it needs that it takes to do this.…We're able to help them troubleshoot as best as we can. Our last resort is to call the company or put in a request [with the manufacturer] to call them.…If we can't find anyone, we will send a letter [communicating] that we have tried multiple times, that their monitoring is out of compliance and either to please send [a transmission] or to call us if they need help. That often works.”

Interviewees also reported calling secondary contacts including patient family members, other caregivers, or home health care providers, as described by this registered nurse from a high performing facility:

“We'll just talk to the spouse or the kids that take care of them….We will then contact their home base primary care nurse or find out the home health agency…and ask them the next time they go into the home if they can help reset the monitor, reconnect, send a transmission.”

#### Addressing persistent nonadherence

Where RM barriers could not be surmounted, some clinicians reported removing patients from RM systems and increasing the frequency of in-person CIED interrogation visits, whereas other clinicians attempted to contact patients indefinitely. Facilities in all performance categories reported using in-person appointments to correct nonadherence through patient education and troubleshooting, with even the suggestion of an increase in the frequency of in-person visits motivating some patients to adhere to RM.

“No, I don't think [we stop trying to address adherence for a veteran patient]. They are always coming in at some point or another and you know if they got an ICD and home monitoring is not going to work for them, sometimes you can get him to comply a little better or get a little more motivated when you tell them, ‘Alright we're going to have to see every three months then instead of once a year.’ They won't want to come every three months. It's a 2-hour drive you know and then they get a little more motivated to work on their home monitoring.”

#### Team communication

High performing facilities reported constant conversation within the device care team and standard operating procedures (covering patient follow-up, review of remote transmissions, device troubleshooting) that eliminated redundant communication with patients, defined team member responsibilities, and guided clinical practice. One registered nurse at a high performing facility described her facility’s process:

“[My nurse colleague and I] have a talk every morning. We just have a really good team approach at contacting these patients.…We just are in constant communication on where we're at with processes and with patients.…We've created swim lanes where we divided up the remote monitoring responsibilities…creating smaller pieces of the pie and giving each person their particular ownership of that remote monitoring process has seemed to help a lot and it doesn't seem so overwhelming.”

#### CIED manufacturer support

Across all performance categories, CIED clinicians valued collaboration with CIED manufacturer representatives for support in patient education, setting up and troubleshooting home monitors, and providing brand-specific expertise for devices. High performing facilities leveraged CIED manufacturer support more often than other facilities by utilizing manufacturer resources as a part of their daily workflow. These facilities referred patients to manufacturer technical support phonelines or called in on behalf of or with patients. They also utilized manufacturer web sites to request manufacturer follow-up with patients and used in-clinic device representative visits for patient education, as one registered nurse stated:

“We do have huge vendor support; they support our providers…and they support our veterans. We have some veterans that live pretty remotely who travel two to three hours to get to our clinic and we have had vendors on multiple occasions go to their homes and help them reconnect and get their remote monitoring set up.…If there's a clinic somewhere that's having a hard time and they're not utilizing their vendors as much as they possibly could, that would really benefit them.”

#### Community care connections

Because many VHA facilities care for patients who have had a CIED implanted outside of VHA, relationships with non-VHA clinicians were essential to ensuring a smooth transition. One registered nurse at a facility that did not implant CIEDs coordinated this often-cumbersome process through formal relationships with non-VHA clinicians:

“[Initial setup RM setup for CIEDs implanted at local hospitals] are usually taken care of by the implanting facility, but we have a really good relationship with the two local [hospitals].…we talk back and forth all the time. [The non-VHA hospital] does an implant. They fax us the implant information right away so we can get them registered. They let us know if [the patient] has been issued a monitor or not.”

## Discussion

Adherence is central to ensuring patients receive the evidence-based benefits from RM of CIEDs, and, consistent with the Class 1A Heart Rhythm Society (HRS) recommendation,^15^^,^^16^ all clinicians in our national study across 26 facilities caring for approximately 15,000 patients with CIEDs within an integrated health care system believed the importance of RM and were motivated to overcome barriers to support patient adherence. Most barriers are in the inner setting subdomain of work infrastructure, a type of structural characteristic concerning issues of organization of tasks and responsibilities within and between individuals and teams. In contrast, facilitators span multiple subdomains of the inner setting, such as information technology infrastructure, and communications. In the innovation domain, we found that consistently reinforcing patient education, performing routine checks for adherence and then immediately addressing nonadherence using protocols, and collaborating with CIED manufacturers helped clinicians support high patient adherence.

Previous studies have documented challenges with maintaining patient RM connectivity and adherence^18^ as well as clinician and patient perceptions regarding RM.^19^ Our findings confirm several results from these studies, including that clinicians trust RM and the importance that clinicians place on patient-centered care and education about RM. Furthermore, our study advances the field by synthesizing successful strategies to support RM adherence through an investigation of the substantial variation in adherence rates across clinics in a nationally integrated health care system. Ensuring that facilities were appropriately staffed was of paramount importance to clinicians, with more than three-fourths communicating the importance of adequate staff to focus on RM. Although limited by a small sample size, our results (Table 3 and Figure 2) demonstrate the relationship between lower patient-to-clinician ratios and higher adherence to RM. High patient-to-clinician ratios can compromise the quality of care when responsibilities are not designated to device clinic staff, particularly in light of increasing unscheduled transmission workload.^16^ Consistent with our findings, the 2023 HRS expert consensus statement on practical management of the remote device clinic acknowledges the centrality of work infrastructure, calling for adequate dedicated staffing that incorporates “a team-based organizational model with formal policies, procedures, and clear definitions of the roles and responsibilities of qualified staff.”^16^

Clinician time requires funding support, which often is not guaranteed. The challenge of demonstrating that RM tasks, such as troubleshooting connectivity and adherence, are revenue-generating sometimes hinders dedicated investment in facility staff who can address them.^18^ Reimbursement considerations are far less salient than patient care in VHA, which awards uniform workload credit for RM, but substantial reorganization of reimbursement schemes, including increased cost transparency,^19^ may be needed in other health systems to adequately support RM activities. Designation of RM adherence as a quality-of-care metric, as the VHA has done through quarterly reports to VHA leadership and a real-time online dashboard viewable by leadership, could also help to increase RM adherence. Novel reimbursement models, such as a bundled annual rate for care of CIED patients,^16^^,^^25^^,^^26^ could help secure adequate staffing so that clinicians can dedicate effort to these tasks.

Despite resourcing challenges, high performing facilities maximize RM adherence through several strategies. In the communication subdomain, formal information sharing and consistent peer collaboration supported by a formal standard operating procedure document can help ensure that often-limited resources to support RM are most efficiently used. This finding is also consistent with the 2023 HRS expert consensus statement, which gives a Class 1 recommendation for clinics to have an established process that includes dedicated staff to facilitate reconnection.^16^ In the information technology subdomain, cloud-based, all-in-one digital dashboards for tracking adherence, especially VANCDSP resources, have facilitated efficient population-level management, as has been done for other cardiovascular conditions.^27^^,^^28^ The VANCDSP hosts these digital dashboards, contacts clinics with lists of patients who are nonadherent, reports adherence as a quality-of-care metric, and sends reminder postcards to patients who have missed a remote transmission,^29^ forming a central RM resource that high performing facilities can utilize to optimize staff effort and efficiently identify clinically urgent needs. An additional possibility would be to centralize RM adherence through a central resource like the VANCDSP; ideally this would be studied, as it would require additional investment and personnel.

Improving patient adherence begins with the patient, and many patients have limited understanding of their CIEDs despite their desire to know and learn more.^30^ Consistent with Class 1 HRS recommendations,^16^ clinicians at facilities with high adherence explained that initiation of RM required repeated, detailed, and individualized patient education before and after CIED implantation. CIED follow-up care also must be tailored to the preferences and needs of individual patients, including addressing technology or privacy concerns as well as challenges such as lack of stable housing. It is likely that more FTE may be needed for facilities to support adherence among patients who have less familiarity with RM and fewer resources; for example, RM adherence is lower among individuals with a lower income.^17^ Clinicians should use a patient-centered approach that communicates CIED follow-up using culturally sensitive information and incorporates patient preferences.^31^ Patient buy-in could be further supported by confirming receipt of remote transmissions and sharing data about device function and parameters.^32^ Finally, in addition to clinician outreach, automatic direct-to-patient notifications for successful or missed transmissions via smartphone notification, home transmitter light-up display, or mailing can support adherence.^33^ Patient family members and caregivers can play a key role driving RM adherence through social support and should be included whenever possible in RM conversations.

High performing clinicians reported that CIED manufacturer support also served as a key resource in clinician and patient education, technical support, and restoration of connectivity; similarly, the 2023 HRS expert consensus recommends manufacturers provide this support.^16^ Because of the variation in RM hardware and function by company, coordinating with knowledgeable manufacturer representatives reduced the troubleshooting burden and technical knowledge requirements for facility staff.

RM adherence will take additional importance to ensure success of newly recommended strategies for patient management, including spacing in-person visits to every 2 years and even possibly stopping routine in-person and remote interrogations in favor of alert-based RM; these will depend heavily on consistent and continuous CIED connectivity.^16^ As remote patient management and remote work options have expanded in the post–COVID-19 health care landscape,^34^ these successful strategies for CIED management, especially effective organization and management of work infrastructure, may also generalize to other areas of care.

### Study limitations

First, we used a purposive sampling approach, and our findings do not include all 122 VHA facilities that provide care for patients with CIEDs or beyond VHA, and findings may differ from non-VHA facilities. Second, we had a small sample size of 26 facilities. However, we interviewed clinicians at facilities around the United States and across different levels of adherence representing a significant portion of VHA, and the lessons for promoting CIED adherence are generalizable to any facility. Third, the interview response rate for facilities with patients in the suboptimal range of RM adherence (100-day adherence <70%) was low (4/14 [29%]) despite 3 attempts to contact them over multiple weeks. Low response rates were likely due to competing priorities and staffing shortages that suggest the challenges of maintaining patient RM adherence. Conversely, high performing facilities were 100% responsive to requests for interviews and surveys, likely due to better staff availability. Future work should seek to use more objective sampling methods of a larger number of clinics, including low-performing sites, in order to determine the most successful strategies for improving RM adherence.

## Conclusion

Using successful strategies from high performing facilities described in this article, amid greater RM utilization^10^ and updated follow-up consensus recommendations,^16^ CIED facilities can optimize often-limited resources, maximizing patient RM adherence and the associated clinical outcome benefits.

## Appendix Supplementary Data

Supplementary Material

ISSM_COREQ_Checklist

## References

[1] Hindricks G., Varma N., Kacet S. (2017). Daily remote monitoring of implantable cardioverter-defibrillators: insights from the pooled patient-level data from three randomized controlled trials (IN-TIME, ECOST, TRUST). Eur Heart J.

[2] Mittal S., Piccini J.P., Snell J., Prillinger J.B., Dalal N., Varma N. (2016). Improved survival in patients enrolled promptly into remote monitoring following cardiac implantable electronic device implantation. J Interv Card Electrophysiol.

[3] Varma N., Piccini J.P., Snell J., Fischer A., Dalal N., Mittal S. (2015). The relationship between level of adherence to automatic wireless remote monitoring and survival in pacemaker and defibrillator patients. J Am Coll Cardiol.

[4] Akar J.G., Bao H., Jones P.W. (2015). Use of remote monitoring is associated with lower risk of adverse outcomes among patients with implanted cardiac defibrillators. Circ Arrhythm Electrophysiol.

[5] Guédon-Moreau L., Kouakam C., Klug D. (2014). Decreased delivery of inappropriate shocks achieved by remote monitoring of ICD: a substudy of the ECOST trial. J Cardiovasc Electrophysiol.

[6] Guedon-Moreau L., Lacroix D., Sadoul N. (2013). A randomized study of remote follow-up of implantable cardioverter defibrillators: safety and efficacy report of the ECOST trial. Eur Heart J.

[7] Ladapo J.A., Turakhia M.P., Ryan M.P., Mollenkopf S.A., Reynolds M.R. (2016). Health care utilization and expenditures associated with remote monitoring in patients with implantable cardiac devices. Am J Cardiol.

[8] Mabo P., Victor F., Bazin P., Ahres S. (2011). COMPAS Trial Investigators. A randomized trial of long-term remote monitoring of pacemaker recipients (The COMPAS trial). Eur Heart J.

[9] Piccini J.P., Mittal S., Snell J., Prillinger J.B., Dalal N., Varma N. (2016). Impact of remote monitoring on clinical events and associated health care utilization: a nationwide assessment. Heart Rhythm.

[10] Holtzman J.N., Wadhera R.K., Choi E. (2020). Trends in utilization and spending on remote monitoring of pacemakers and implantable cardioverter-defibrillators among Medicare beneficiaries. Heart Rhythm.

[11] Perl S., Stiegler P., Rotman B. (2013). Socio-economic effects and cost saving potential of remote patient monitoring (SAVE-HM trial). Int J Cardiol.

[12] Varma N., Love C.J., Michalski J., Epstein A.E. (2021). Alert-based ICD follow-up: a model of digitally driven remote patient monitoring. JACC Clin Electrophysiol.

[13] Timmermans I., Meine M., Szendey I. (2019). Remote monitoring of implantable cardioverter defibrillators: patient experiences and preferences for follow-up. Pacing Clin Electrophysiol.

[14] Ricci R.P., Morichelli L., Quarta L. (2010). Long-term patient acceptance of and satisfaction with implanted device remote monitoring. Europace.

[15] Slotwiner D., Varma N., Akar J.G. (2015). HRS expert consensus statement on remote interrogation and monitoring for cardiovascular implantable electronic devices. Heart Rhythm.

[16] Ferrick A.M., Raj S.R., Deneke T. (2023). 2023 HRS/EHRA/APHRS/LAHRS expert consensus statement on practical management of the remote device clinic. Heart Rhythm.

[17] Muniyappa A.N., Raitt M.H., Judson G.L. (2022). Factors associated with remote monitoring adherence for cardiovascular implantable electronic devices. Heart Rhythm.

[18] Harvey M., Seiler A. (2022). Challenges in managing a remote monitoring device clinic. Heart Rhythm O2.

[19] Fraiche A.M., Matlock D.D., Gabriel W., Rapley F.A., Kramer D.B. (2021). Patient and provider perspectives on remote monitoring of pacemakers and implantable cardioverter-defibrillators. Am J Cardiol.

[20] Ottenberg A.L., Swetz K.M., Mueller L.A., Gerhardson S., Mueller P.S. (2013). "We as Human Beings Get Farther and Farther Apart": the experiences of patients with remote monitoring systems. Heart Lung.

[21] Damschroder L.J., Reardon C.M., Opra Widerquist M.A., Lowery J. (2022). Conceptualizing outcomes for use with the Consolidated Framework for Implementation Research (CFIR): the CFIR Outcomes Addendum. Implement Sci.

[22] Damschroder L.J., Aron D.C., Keith R.E., Kirsh S.R., Alexander J.A., Lowery J.C. (2009). Fostering implementation of health services research findings into practice: a consolidated framework for advancing implementation science. Implement Sci.

[23] (2022).

[24] Guest G., Namey E., Chen M. (2020). A simple method to assess and report thematic saturation in qualitative research. PLoS One.

[25] Yang S., Stabenau H.F., Kiernan K., Diamond J.E., Kramer D.B. (2023). Clinical utility of remote monitoring for patients with cardiac implantable electrical devices. J Interv Card Electrophysiol.

[26] Mecklai K., Smith N., Stern A.D., Kramer D.B. (2021). Remote patient monitoring — overdue or overused?. N Engl J Med.

[27] Dorsch M.P., Chen C.S., Allen A.L. (2022). Nationwide implementation of a population management dashboard for monitoring direct oral anticoagulants: insights from the Veterans Affairs Health System. Circ Cardiovasc Qual Outcomes.

[28] Foster M., Albanese C., Chen Q. (2020). Heart failure dashboard design and validation to improve care of veterans. Appl Clin Inform.

[29] McLaughlin M., Raitt M.H., Tarasovsky G., Dhruva S.S. (2023). Informational postcards improve access to remote monitoring among veterans with pacemakers and implantable cardioverter-defibrillators: a stepped-wedge randomized controlled trial. J Am Coll Cardiol.

[30] Patel D., Hu P., Hilow H. (2020). The gap between what patients know and desire to learn about their cardiac implantable electronic devices. Pacing Clin Electrophysiol.

[31] Rosman L. (2019). Remote monitoring of implantable cardioverter defibrillators: aligning patient preferences and provider recommendations. Pacing Clin Electrophysiol.

[32] Slotwiner D.J., Tarakji K.G., Al-Khatib S.M. (2019). Transparent sharing of digital health data: a call to action. Heart Rhythm.

[33] Petersen H.H., Larsen M.C.J., Nielsen O.W., Kensing F., Svendsen J.H. (2012). Patient satisfaction and suggestions for improvement of remote ICD monitoring. J Interv Card Electrophysiol.

[34] Varma N., Marrouche N.F., Aguinaga L. (2021). HRS/EHRA/APHRS/LAHRS/ACC/AHA worldwide practice update for telehealth and arrhythmia monitoring during and after a pandemic. Europace.

