# Supplementary material for: Strategies to enhance remote monitoring adherence among patients with cardiovascular implantable electronic devices
Source: Heart Rhythm O2. 2023 Nov 8;4(12):794–804. doi: 10.1016/j.hroo.2023.11.002 (PMC10774668; doi:10.1016/j.hroo.2023.11.002)
Supplement: Supplementary Material [file mmc1.docx]

**SUPPLEMENTAL MATERIAL**

Detailed Methods

Supplemental Methods includes the survey instrument and interview guide used to gather data from clinicians caring for patients with cardiovascular implantable electronic devices (CIEDs). These data were gathered as part of a larger study. Questions that elicited results relevant to the current study are bolded.

Survey Instrument

This survey asks about your experience with remote monitoring adherence within VA and perspectives about tools to help with supporting remote monitoring adherence.

Section 1

Informational Questions about You

1. **Please tell us your title within your VA clinic**

**<Required to answer. Single choice.>**

1. **DO/MD**
2. **Advanced Practice Provider (APP)**
3. **RN**
4. **Medical Instrument Technician (MIT)**
5. **Medical Support Assistant (MSA)**

**2.How many years have you been working with the VA device clinic?**

**<Required to answer. Single choice.>**

1. **<1 year**
2. **1-5 years**
3. **6-10 years**
4. **10+ years**

**3.When do you address remote monitoring adherence (making sure patients transmit when they are supposed to and troubleshooting missed transmissions)?**

**<Required to answer. Single choice.>**

1. **Never**
2. **Just when I see patients in clinic and check to see if they have transmitted**
3. **I set aside some time every day/week/month to look at people that have not transmitted and try to get them transmitting**
4. **Other**

**4.On average, approximately how many hours/week do you spend focused on remote monitoring adherence (making sure patients transmit when they are supposed to and troubleshooting missed transmissions)?**

**<Required to answer. Single line text.>**

- **Please enter a number less than or equal to 40**

5.On average, how many hours/week do you spend using the NCDSP Dashboard?

<Required to answer. Single line text.>

- Please enter a number less than or equal to 40

6.Since your clinic has had access to the NCDSP Dashboard, how many (if any) additional hours/week do you now devote to Veteran adherence to remote monitoring, compared to what you had before the dashboard became available?

<Single line text.>

- Please enter a number less than or equal to 40

7.On average, how many hours/week do you spend using the NCDSP Patient Registration Application?

<Required to answer. Single line text.>

- Please enter a number less than or equal to 40

8.Within your VA clinic, are there any other individuals who routinely spend time on remote monitoring adherence? Please check all that apply.

<Required to answer. Multiple choice.>

1. DO/MD
2. Advanced Practice Provider (APP)
3. RN
4. Medical Instrument Technician (MIT)
5. Medical Support Assistant (MSA)

Section 2

Perspectives on Remote Monitoring Adherence and Tools to Support Adherence

**9.How important is remote monitoring adherence to improving outcomes for patients with CIEDs?**

**<Required to answer. Likert.>**

1. **Not at all important**
2. **Slightly important**
3. **Important**
4. **Very important**
5. **Extremely important**

**10.What tools do you use to support remote monitoring adherence among Veterans in your clinic?**

**If you select "other," please provide additional details.**

**<Required to answer. Multiple choice.>**

1. **NCDSP Dashboard**
2. **NCDSP Patient Registration Application**
3. **Monitor company sites**
4. **Other**

11.Has availability of the NCDSP Dashboard increased the importance placed on remote monitoring adherence by your clinic?

<Required to answer. Single choice.>

1. Yes
2. No

12.How helpful do you find the following for remote monitoring adherence?

<Required to answer. Likert.>

1. Not at all helpful
2. Slightly helpful
3. Somewhat helpful
4. Very helpful
5. Extremely helpful

<Categories>

1. NCDSP Dashboard (OVERALL)
2. NCDSP Patient Registration Application (OVERALL)
3. Overall adherence score (% of patients who have sent a transmission within the past 100 or 200 days)
4. Ability to sort by time since last transmission
5. Patient's generator and lead information
6. NCDSP interpretation of remote transmission
7. PDF of remote transmission

**13.Which do you prefer to support your remote monitoring adherence efforts?**

**If you select "other," please provide additional details.**

**<Required to answer. Single choice.>**

1. **NCDSP Dashboard**
2. **NCDSP Patient Registration Application**
3. **Both**
4. **Other**

**14.When do you generally contact patients about a missed transmission?**

**<Required to answer. Single choice.>**

1. **< 1 month after missed transmission**
2. **1 to 3 months after missed transmission**
3. **4 to 6 months after missed transmission**
4. **7 to 12 months after missed transmission**
5. **I usually do not contact patients who have missed transmissions**

Section 3

Demographic Questions

**15.Please provide your age (years).**

**<Required to answer. Single choice.>**

1. **<30**
2. **31-40**
3. **41-50**
4. **51-60**
5. **61-70**
6. **71 or older**

**16.How many years have you worked within VA?**

**<Required to answer. Single choice.>**

1. **<1 year**
2. **1-5 years**
3. **6-10 years**
4. **>10 years**

**17.Please provide your gender self-identification**

**<Required to answer. Single choice.>**

1. **Woman**
2. **Man**
3. **Transgender woman**
4. **Transgender man**
5. **Non-binary**
6. **Other**
7. **Prefer not to say**

**18.Please provide your racial and ethnic self-identification**

**<Required to answer. Multiple choice.>**

1. **American Indian or Native American**
2. **Asian**
3. **Black or African American**
4. **Hispanic, Latinx, or of Spanish origin**
5. **Pacific Islander**
6. **White**
7. **Other**
8. **Prefer not to say**

**19. Are there other individuals at your cardiology device clinic who are focused on remote monitoring adherence efforts with whom you think we should also speak? If so, please provide their email address. (OPTIONAL)**

**<Single line text.>**

- **Enter your answer**

Interview Guide

Introduction

Thank you again for taking time out of your busy schedule to speak with us.

Before we get started, I wanted to give you some more background on the project and then see if you have any questions. This quality improvement study is being conducted by the Measurement Science Quality Enhancement Research Initiative (QUERI) in collaboration with National Cardiac Device Surveillance Program (NCDSP) to understand facilitators and barriers to remote monitoring adherence and best inform how efforts to improve adherence can be supported.

With your permission, we will be recording the audio from this interview and using the Teams transcription feature to keep a record for analysis as well as taking notes. As a reminder, you can skip any question that you don’t want to answer. However, your identity will be kept confidential and nothing you say will be able to be traced back to you. The interviews can last up to an hour – does that still work with your schedule?

Do you have any questions for me before we get started?

Okay, I am turning on the recording.

Rapport-Building Questions

To start off, please tell me your title and briefly describe your role at [site].

How long have you been in that role and how long have you been with the VA?

Best Practices (ask all participants)

**Q1 How is remote monitoring adherence a priority for your clinic? (Inner Setting)**

*Probes:*

- *Are there strategic goals around remote monitoring at your clinic or within your Veterans Integrated Service Network (VISN)? (planning)*
- *Are the remote monitoring adherence metrics in the specialty care dashboard or the specialty care quarterly report discussed within your device clinic? (Goals and Feedback)*
- *Is there any particular person or group championing remote monitoring? (engaging)*
- *Was this champion based at your clinic or from outside your clinic? (intervention source)*
- *Is addressing remote monitoring adherence incentivized or rewarded? (incentives and rewards) How so?*
- *Are leaders at your clinic invested in remote monitoring? (leadership engagement, implementation climate) How so?*
- *Taking us back to when you started at the device clinic, what has changed since then in terms of remote monitoring strategies or workflow?*

**Q2) Do you implant CIEDs at your clinic? How do you initiate remote monitoring for veterans?**

*Probes:*

- *How are monitors given to Veterans distributed and activated? How does this differ by CIED manufacturer?*
- *What information are Veterans provided about remote monitoring? Why provides this information and when?*
- *When in their care is remote monitoring first discussed with Veterans?*
- *When in their care are Veterans provided with a remote transmitter?*

**Q3) How do you monitor adherence of individual patients? (If answer is “I/we don’t monitor,” move to Q7)**

*Probes:*

- *Are there relative advantages of those approaches used for remote monitoring? (relative advantage)*
- *How do those approaches meet clinician needs and patient needs? (Patient Needs and Resources)*
- *Do you use the NCDSP PowerBI Dashboard? [If results from Survey Instrument are unclear]*

Only asked of clinicians using the dashboard

Q4) Why are you using the NCDSP Dashboard? How does it help you?

*Probes:*

- *Better than other tools for remote monitoring (relative advantage)*
- *Easy to use (complexity)*
  - *Easier to use than manufacturer websites?*
  - *Combined information in one place? Which pieces of information?*
- *Directed to use it (leadership engagement)*
- *Access to knowledge (many features)*
  - *Quickly gathering information for manufacturer representatives?*
  - *Pulling data for patient management?*

Q5) How do you use the NCDSP Dashboard?

*Probes:*

- *How often do you use the dashboard? (per day/week/month)*
- *Patient visit/communication preparation?*
- *Determine if a new patient has never been enrolled in remote monitoring?*
- *Spot check for NCDSP reviewer comments?*
- *Call patients? (yes/no)*
- *Use it to write notes in Computerized Patient Record System (CPRS)? (yes/no)*
- *Other (list)*

Only asked of clinicians not using the dashboard

Q6) Why are you not using the NCDSP dashboard?

*Probes:*

- *Own superior system for tracking patients and adherence? (relative advantage) What is it?*
- *Dashboard too complex? (complexity) How so?*
- *Lack time? (relative priority)*
- *Remote monitoring adherence not a priority in clinic? (culture)*

**Q7) How do you address remote monitoring non-adherence / missed transmissions?**

*Probes:*

- *What methods do you use to address non-adherence with patients? What is the order in which you address them?*
  - *Call patients?*
  - *Check patients for adherence at in-person evaluations? How do you perform these checks?*
  - *Send patients letters? What are the source of those letters?*
  - *Are any of these communications automated?*
- *Do you involve the companies in troubleshooting? If so, how?*
- *Are family members engaged to help with remote monitoring adherence? Home healthcare workers? How and when are they engaged?*
- *At what point do you stop trying to address nonadherence (if ever)?*

*Probes for those who do not monitor and were redirected from question 3:*

- *Why don’t you monitor remote monitoring adherence?*
  - *Lack of resources? If so, what specific resources would be necessary?*
  - *Feel it is unnecessary? Feel that patients should come for in-person evaluations?*
- *How do you care for your CIED patients who do not participate in remote monitoring?*

Only asked of high-performing VA clinics (i.e., high adherence)

**Q8) Why do you think that your strategies for maintaining adherence are successful?**

*Probes:*

- *Own superior system for tracking patients and adherence? (relative advantage) What is it?*
- *Dedicated staff?*
- *Are there any materials that you use to communicate with Veterans that you would be willing to disseminate within VA?*
- *Does your clinic have an standard operating procedure (SOP) for remote monitoring adherence? If so, would you be willing to share it with us? Can we share it with other clinics?*

Wrap-up: ask all participants

**Q9) How can improvement in Veteran adherence to remote monitoring be supported in your VA clinic?**

*Probes:*

- *More data about benefits of remote monitoring? (evidence strength and quality)*
- *Substitution of in-person visits? (relative advantage)*
- *More support within your clinic? (relative priority)*
- *More support from companies? (external policies and incentives)*
- *Better information to patients? (knowledge and beliefs, self-efficacy to transmit)*
- *More support from other sources? If yes, which sources?*

Q10) How many total staff members are there in your clinic? (MD/DO, APP, RN, MIT, MSA)

- Of those people you mentioned, how many hours per week do they spend on remote monitoring adherence?

Supplemental Tables

**Supplemental Table 1: Facility Category of Adherence to Remote Monitoring.**

| **Facility Performance Category** | **n=26** | **Remote Monitoring Adherence Category** |
| --- | --- | --- |
| High-Performing | 8 | ≥ 90% of patients with a CIED followed by the facility had sent a remote transmission in the past 100 days |
| Normal to Target Performing | 14 | 70-89.9% of patients with a CIED followed by the facility had sent a remote transmission in the past 100 days |
| Suboptimal Performing | 4 | <70% of patients with a CIED followed by the facility had sent a remote transmission in the past 100 days |

CIED = cardiovascular implantable electronic device

Supplemental Table 2: 100- and 200-Day Adherence by VA Facility Size

| **Facility Size (n=26)** | **100 Day Adherence %** | **200 Day Adherence %** |
| --- | --- | --- |
| >1000 patients | | |
| 1 | 96% | 99% |
| 2 | 84% | 89% |
| 3 | 84% | 89% |
| 4 | 80% | 85% |
| 500-999 patients | | |
| 5 | 92% | 96% |
| 6 | 91% | 95% |
| 7 | 91% | 95% |
| 8 | 90% | 96% |
| 9 | 89% | 97% |
| 10 | 80% | 83% |
| 11 | 76% | 82% |
| 12 | 74% | 79% |
| 13 | 70% | 74% |
| 250-499 patients | | |
| 14 | 94% | 97% |
| 15 | 92% | 95% |
| 16 | 89% | 95% |
| 17 | 85% | 91% |
| 18 | 80% | 86% |
| 19 | 77% | 82% |
| 20 | 66% | 70% |
| <250 patients | | |
| 21 | 92% | 95% |
| 22 | 84% | 91% |
| 23 | 83% | 91% |
| 24 | 65% | 76% |
| 25 | 57% | 62% |
| 26 | 46% | 49% |

**Supplemental Table 3: Clinician Characteristics**

| Characteristic | High-Performing  Facility  (n=9) | Normal to Target Performing  Facility  (n=12) | Suboptimal Performing  Facility  (n=3) | **Total**  **(n=24)** |
| --- | --- | --- | --- | --- |
| Title |  |  |  |  |
| APP | 3 | 4 | 0 | **7** |
| MIT | 2 | 2 | 1 | **5** |
| RN | 4 | 6 | 2 | **12** |
| Time worked with VHA device clinic |  |  |  |  |
| <1 year | 1 | 2 | 0 | **3** |
| 1-5 years | 3 | 5 | 2 | **10** |
| 6-10 years | 2 | 4 | 1 | **7** |
| 10+ years | 3 | 1 | 0 | **4** |
| Time worked within VHA |  |  |  |  |
| <1 year | 0 | 1 | 0 | **1** |
| 1-5 years | 4 | 3 | 1 | **8** |
| 6-10 years | 1 | 2 | 2 | **5** |
| >10 years | 4 | 6 | 0 | **10** |
| Age (years) |  |  |  |  |
| 31-40 | 2 | 2 | 0 | **4** |
| 41-50 | 2 | 5 | 3 | **10** |
| 51-60 | 5 | 4 | 0 | **9** |
| 61-70 | 0 | 1 | 0 | **1** |
| Gender self-identification |  |  |  |  |
| Man | 1 | 5 | 1 | **7** |
| Woman | 8 | 7 | 2 | **17** |
| Racial and Ethnic self-identification (check all that apply) |  |  |  |  |
| Asian | 0 | 1 | 1 | **2** |
| Black or African American | 0 | 0 | 1 | **1** |
| Hispanic, Latinx, or of Spanish origin | 1 | 0 | 0 | **1** |
| Pacific Islander | 0 | 1 | 0 | **1** |
| White | 9 | 8 | 3 | **20** |
| Other | 0 | 0 | 1 | **1** |
| Prefer not to say | 0 | 2 | 0 | **2** |
| Region |  |  |  |  |
| Continental | 0 | 3 | 0 | **3** |
| Midwest | 6 | 4 | 0 | **10** |
| Northeast | 0 | 0 | 1 | **1** |
| Pacific | 3 | 2 | 1 | **6** |
| Southeast | 0 | 3 | 1 | **4** |
| Clinic size (patient population) |  |  |  |  |
| >1000 | 1 | 3 | 0 | **4** |
| 500-999 | 5 | 3 | 0 | **8** |
| 250-499 | 2 | 4 | 0 | **6** |
| <250 | 1 | 2 | 3 | **6** |

APP = Advance Practice Provider; MIT = Medical Instrument Technician; RN = Registered Nurse; VHA = Veterans Health Administration

**Supplemental Table 4: Results of Spearman’s Correlation with Patient Adherence to Remote Monitoring**

| **Measure (df =18)** | **Spearman correlation coefficient** | **p-value** |
| --- | --- | --- |
| Total FTE | 0.53* | 0.019 |
| FTE Spent on Remote Monitoring | 0.25 | 0.283 |
| FTE Spent on Other Device-Specific Care of Veterans with CIEDs | 0.67* | 0.002 |

FTE = Full-Time Equivalent

CIEDs = cardiovascular implantable electronic devices

* Statistically significant result (p <0.05)
